# Survival of patients with 2014 FIGO stage IIIC high-grade serous ovarian cancer who were treated with platin-based adjuvant chemotherapy

**DOI:** 10.55730/1300-0144.5980

**Published:** 2025-03-06

**Authors:** Mehmet ÜNSAL, Meltem YÜCESOY, Okan OKTAR, Yeşim UÇAR, Gülşah TİRYAKİ GÜNER, Okan AYTEKİN, A. Alp TOKALIOĞLU, Hande Esra KOCA YILDIRIM, Fatih KILIÇ, Burak ERSAK, Caner ÇAKIR, Dilek YÜKSEL, Çiğdem KILIÇ, Sevgi KOÇ, Günsu KİMYON CÖMERT, Özlem MORALOĞLU TEKİN, Yaprak ENGİN ÜSTÜN, Taner TURAN

**Affiliations:** 1 Division of Gynecologic Oncology, Department of Obstetrics and Gynecology, Faculty of Medicine, Health Sciences University, Ankara City Hospital, Ankara, Turkiye; 2 Division of Gynecologic Oncology, Department of Obstetrics and Gynecology, Faculty of Medicine, Health Sciences University, Etlik Zübeyde Hanim Women’s Health Training and Research Hospital, Ankara, Turkiye

**Keywords:** Stage IIIC, high-grade serous ovarian cancer, survival, cytoreduction

## Abstract

**Background/aim:**

Epithelial ovarian cancer is the second most prevalent type of gynecological cancer. We aimed to investigate prognostic factors related to survival in patient with 2014 FIGO stage IIIC high-grade serous ovarian cancer (HGSOC).

**Materials and methods:**

Two hundred fifty eight patients were evaluated retrospectively. The absence of a visible tumor was determined as maximum cytoreduction, and a residual tumor size of 1 cm or less was determined as optimal cytoreduction. Patients who underwent cytoreduction followed by a combination of platinum and taxane adjuvant chemotherapy were included. Exclusion criteria for the study were patients taking neoadjuvant chemotherapy and those with suboptimal surgery.

**Results:**

Optimal cytoreduction was performed in 107 and maximal cytoreduction was performed in 151 patients. The five-year PFS rate was 27% and the five-year DSS rate was 76%. While high ascites volume and optimal cytoreduction were identified as independent prognostic factors for disease failure, only optimal cytoreduction was an independent prognostic factor for survival.

**Conclusion:**

The maximal cytoreduction is improves PFS and DSS in patients with 2014 FIGO stage IIIC HGSOC as our study results. Therefore, maximum surgical effort and radical cytoreductive procedures should be performed.

## 1. Introduction

Epithelial ovarian cancer (EOC) is the 2nd most prevalent type of gynecological cancer [1]. Different histologic subtypes of EOC are defined but the serous type is the most common and accounts for 88% of stage III–IV disease [2]. Because patients usually do not exhibit specific symptoms at an early stage, ovarian malignancies are typically identified as an advanced disease.

High-grade serous ovarian cancer (HGSOC), fallopian tube cancer, and peritoneal cancers are grouped similarly in the 2014 International Federation of Gynecology and Obstetrics (FIGO) staging system. Stage I of the disease is limited to one ovary or fallopian tube, and Stage II is restricted to the region below the pelvic brim. Stage IV was defined as a distant metastasis excluding peritoneal metastasis. Contrary to the 1988 FIGO staging system, in which regional lymph node metastasis was defined as stage IIIC, positive lymph node metastasis changed to stage IIIA in the new classification. Stage IIIC, characterized by widespread metastases of tumors larger than 2 cm in the upper abdomen, is associated with the lowest survival rate [3].

The standard treatment involves a surgical cytoreduction, afterwards adjuvant chemotherapy with platinum-based drugs. The five-year survival rates for stage 3–4 ovarian cancers have been found to be 20%–30% [4]. The presence of tumors in the upper abdomen, postoperative residual tumor size and number of residual tumor are suggested to be the main prognostic factors in advanced-stage ovarian cancer [5, 6].

Survival and related factors are not well defined in the limited available studies because of the heterogeneity of stage IIIC HGSOC. The aim of current study is to investigate prognostic factors related to survival in stage IIIC HGSOC.

## 2. Materials and methods

Two hundred fifty eight patients who were diagnosed as having stage IIIC HGSOC according to the 2014 FIGO staging system were evaluated retrospectively. Patients who underwent cytoreduction followed by a combination of platinum and taxane adjuvant chemotherapy from January 1993 to April 2020 were enrolled in the study. Patients with synchronous or secondary malignancies, those with non-serous cell and non-epithelial components, patients who received neoadjuvant chemotherapy or non-platinum-based adjuvant chemotherapy, except for FIGO 2014 stage IIIC, patients who underwent suboptimal surgery or surgery in other centers were excluded. All surgical procedures were performed by experienced gynecologic oncologists. The study was approved by the national ethics committee (Number: 90057706-799).

The demographic characteristics of the patients, intraoperative findings, postoperative pathology reports, adjuvant chemotherapy regimens, and oncologic outcomes were retrospectively harvested from the electronic medical record system, from the patients’ files.

All patients underwent an extrafascial hysterectomy with bilateral salpingo-oophorectomy, total omentectomy, systematic lymphadenectomy, and cytoreductive surgery as standard procedures. In the presence of macroscopic tumoral infiltration, parietal or visceral peritonectomy, and diaphragm stripping were performed in addition to standard staging surgery. The absence of a visible tumor was determined as maximum cytoreduction, and a residual tumor size of one centimeter or less was determined as optimal cytoreduction. Suboptimal cytoreduction was determined as a residual tumor size greater than one centimeter.

FIGO 2014 staging criteria were used. Response to chemotherapy was assessed according to the Response Evaluation Criteria in Solid Tumors (RECIST 1.1) criteria. Anamnesis, physical examination, tumor markers like CA-125, and imaging modalities like CT or MRI were used to evaluate the chemotherapy response 30 days after the last dose of the adjuvant chemotherapy. A complete clinical response was characterized as the absence of any visible macroscopic tumor, whereas a partial clinical response was considered as a reduction in macroscopic tumor size by more than 50%. Macroscopic tumor size was decreased by less than fifty percent or increased by less than twenty-five percent, which was determined to be a stable disease. Macroscopic tumor size increased by more than 25%, and or finding a new macroscopic tumor was defined as a progressive disease [7].

Refractory disease was determined as the disease progression during first-line adjuvant chemotherapy. In cases of partial clinical response and stable disease, the same chemotherapy methods were continued. During this adjuvant chemotherapy, the patients were reassessed, and a final categorization resulted in either refractory disease or complete clinical response. An increased tumor marker (CA-125) and the identification of new lesions using radiologic methods in individuals who had a complete clinical response were defined as recurrent diseases. “Disease failure” was a term to describe platinum-refractory and recurring disease. After patients had a complete clinical response, routine surveillance was performed. This included pelvic examinations, complete blood counts, tumor markers, blood chemistry, and abdominal-pelvic ultrasonography. Also, every year, chest X-rays were conducted.

### 2.1. Statistical analysis

For continuous variables, descriptive statistics are represented as mean ± standard deviation or median (min–max); for categorical variables, they are expressed as numbers and percentages. For the categorical parameters, the Chi-square test was employed, and for the continuous parameters, the analysis of variance (ANOVA) table test was utilized. We used the Kaplan-Meier method to assess PFS and DSS. Log-rank test was used to compare survival rates. A p-value of less than 0.05 was the threshold for statistical significance.

A multivariate analysis model was created with variables that were statistically significant in univariate analysis and performed using the Cox proportional hazards model to evaluate independent factors that affected survival. The Statistical Package for Social Sciences (IBM SPSS Inc., Chicago, IL, USA) version 20.0 software program was used to conduct the statistical analyses.

## 3. Results

At diagnosis, the patients’ ages ranged from 23 to 80 years old, with a mean of 52.2 ± 9.8 years. A total of 57 (3–160) lymph nodes were removed. In a total of 200 (77.5%) patients with lymph node metastasis, only pelvic lymph nodes were metastatic in 15, only paraaortic lymph nodes were in 52, and both were positive in 124 patients. Metastatic lymph node site not reported in 9 patients. The median metastatic lymph node count was 9 (1–88). The median preoperative CA-125 level was 525 (3–25.000) IU/mL, and the median ascites volume was 2000 cc (50-18.000). In 175 patients (67.8%), peritoneal cytology was positive, and in 227 patients (88%), omental metastasis was found. Optimal surgery was performed in 107 and maximal surgery was performed in 151 patients. Clinicopathologic characteristics are detailed in Table 1.

### 3.1. Survival analysis

Two hundred and thirty-five (91.1%) of the patients had a complete clinical response after adjuvant treatment. Forty-point-five (3–241) months was the median follow-up duration. During this period, 164 (63.6%) patients of the total study group were defined as the disease failure group, which consisted of 23 (8.9%, n=23/258) patients with platinum-refractory disease, and 141 (54.7%, n = 141/235) patients with observed recurrence. The five-year PFS rate was 27% and the five-year DSS rate was 76%.

**Figure 1:**
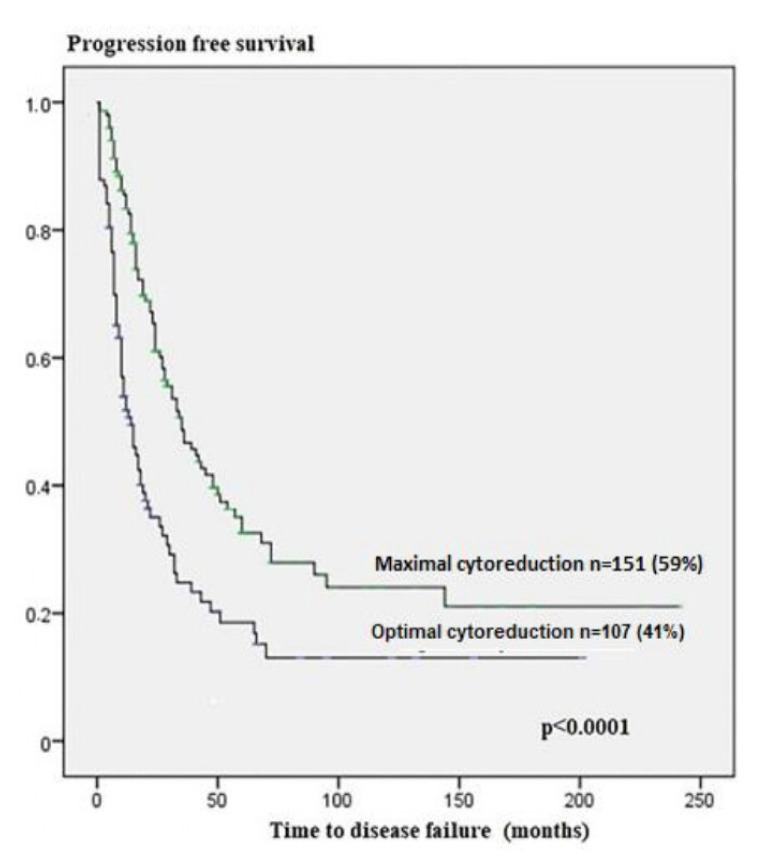
The relationship between type of cytoreduction and progression free survival analysis.

Univariate analysis revealed that peritoneal cytology, omental metastasis, ascites volume, and cytoreduction were associated with PFS. Ascites volume (odds ratio (OR) = 2.080, 95% confidence intervals (CI): 1.299–3.328; p = 0.002 and optimal cytoreduction (OR = 1.735, 95% CI: 1.083–2.777; p = 0.022) were found to be independent prognostic factors of disease failure in multivariate analysis (Figure 1) (Table 2).

**Figure 2:**
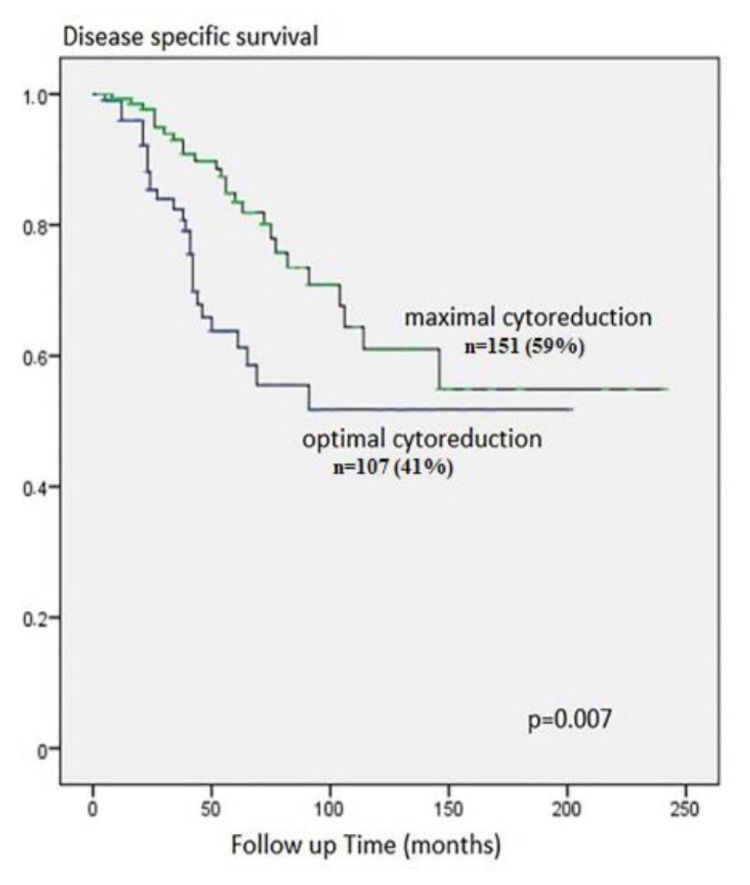
The relationship between type of cytoreduction and disease specific survival analysis.

According to univariate analysis, DSS was associated with uterine serosal invasion, omental metastasis, ascites volume, and outcome of cytoreduction. In multivariate analysis, only optimal cytoreduction, which was found to be an independent risk factor, was statistically significant for death because of disease (OR = 2.257, 95% CI: 1.039–4.904; p *=* 0.040) (Figure 2) (Table 3).

Cytoreduction outcomes determined the complete clinical response after adjuvant chemotherapy. In a total number of 151 patients who underwent maximal cytoreduction, 145 (96%) patients achieved complete clinical response after adjuvant chemotherapy. However, in the optimal cytoreduction group (107 patients), complete clinical response was evaluated in 90 (84.1%) patients. The maximal and optimal cytoreduction groups’ platinum-refractory disease rates were 4% and 15.9%, respectively (p = 0.001). In addition, while the probability of recurrence was 55.2% in maximal cytoreduction, it was 67.8% in optimal cytoreduction (p *=* 0.055).

## 4. Discussion

We investigated the prognostic parameters of 258 individuals with stage IIIC HGSOC in this retrospective research. Cytoreduction outcomes and ascites volume were defined as independent risk factors for PFS. In the optimally surgically debulked group, disease failure was significantly higher than in the maximally debulked group (OR = 1.735). However, only cytoreduction outcomes were defined as an independent risk factor for DSS. Death because of disease in the optimally debulked group was more than two-fold higher compared with the maximally debulked group (OR = 2.257). Our data also showed that high ascites volume, one of the two factors related to PFS with cytoreduction, was significant in multivariate analysis (OR = 2.080). The likelihood of developing platinum-refractory disease was significantly higher in the optimal cytoreduction group.

Eisenhauer et al. analyzed 262 patients with stage IIIC or IV disease who undergone cytoreduction. The authors reported improved rates of response to initial chemotherapy, lower rates of platinum resistance, and improved PFS and overall survival in the maximal cytoreduced group compared with patients in the optimally cytoreduced group [8]. Luyckx et al. published a study on 527 patients diagnosed with FIGO stage IIIC and IV HGSOC who had cytoreduction. Complete cytoreduction was found as the most significant independent factor affecting both DFS and OS in that study. Residual disease, timing of surgery, and types of surgery were found to be independent factors influencing disease-free survival. For overall survival, only residual disease was found to be significant [9].

Confirming our outcomes, Aletti et al. reported that in multivariate analyses, only residual disease was an independent factor predicting overall survival in a total of 194 patients with stage IIIC HGSOC. Overall survival was significantly longer in the patient group who underwent surgery by experienced oncologists [10]. Chi et al. found that the significant prognostic markers were patient age, the presence of ascites, and the size of residual disease [11]. From 2004 to 2009, with the use of more extensive surgical procedures, the rate of optimal debulking surgery increased from 50% to 80% (p *<* 0.001); and median overall survival improved from 43 months to 54 months [12]. Also, over time, with increased aggressive surgical implementation for cytoreduction to obtain no visible tumor after surgery, better oncologic outcomes were demonstrated in existing data [10, 13, 14].

Stage and residual volume of tumor after primary cytoreduction are the most consistently reported prognostic factors [15, 16]. With the intent to improve survival, numerous studies were designed to define the cut-off diameter of the residual tumor with varying sizes of 0.5–2 cm being regarded as ‘optimal’ after cytoreduction. In their meta-analysis involving 6962 patients with advanced ovarian carcinoma, Bristow et al. suggested that each 10% increase in the proportion of optimal cytoreduction including small residual disease was associated with a 5.5% increase in median survival time [17]. The Gynecologic Oncology Group (GOG) defined ≤1 cm in tumor diameter as ‘optimal’ in their prospective study [18]. Even though there is a tendency to accept ≤1 cm diameter in residual tumor size as optimal in studies, leaving any macroscopically visible tumor after cytoreduction is no longer a desirable surgical outcome [19].

Gasimli et al. reported 218 patients with primary ovarian cancer who received maximal cytoreduction. FIGO 1988 stage IIIC due to only lymph node involvement has better survival compared with peritoneal implants over 2 cm. In addition, survival was similar among patients who were lymph node-negative and positive with existing peritoneal implants over 2 cm [20]. Cliby et al. demonstrated similar outcomes in their small study group and emphasized the necessity for not stratifying patients classified as having Stage IIIC disease based solely on nodal disease when comparing outcomes [21]. In our study, according to multivariate analysis, lymph node metastasis showed no significant difference in either PFS or DSS.

As with most studies based on cytoreduction on ovarian cancer, the retrospective nature of our study is considered a limitation. We aimed to ensure randomization by including the whole cohort in the study to reduce the possible risk of bias because of the single-center and retrospective nature of our study. Secondly, the number, size and distribution of residual tumors were unknown in the optimal surgery group. Previous studies’ patient populations consisted of mostly stage IIIC and stage IV regarded as having advanced ovarian cancer. In the few studies that investigated prognostic factors of FIGO 2014 stage IIIC HGSOC, patients who underwent maximal, optimal, and suboptimal debulking with different pathologic tumor types comprised the study populations. Factors related to DSS and PFS are not well defined because of the heterogeneity of parameters such as lymph node positivity, revised staging, and the type of cytoreductive surgery. In that respect, our study group is more homogenous compared with previous data. Also, all surgical procedures were performed by experienced gynecologic oncologists, likewise, all pathologic materials were assessed by pathologists specialized in gynecology.

Our study showed that maximal cytoreduction is correlated with prolonged DSS and PFS in patients with 2014 FIGO stage IIIC HGSOC. Similarly, because the likelihood for recurrence and development of platinum-refractory disease are significantly decreased in maximal cytoreduction, resection of all visible tumors must be the main goal of cytoreduction. In conclusion, aggresive cytoreductive procedures should be performed for maximal cytoreduction.

## Figures and Tables

**Table 1:** Features of entire cohort.

Parametre		Mean±SD	Median (range)
Age at initial diagnosis (years)		52.2 ± 9.8	50 (23–80)
Preoperative CA 125 (IU/mL)		1602 ± 2925	525 (3–25.000)
Total removed lymph node count		58 ± 26.1	57 (3–160)
Pelvic removed lymph node count		38.8 ± 17.9	37 (2–112)
Paraaortic removed lymph node count		23.1 ± 11.2	23 (3–54)
Total metastatic lymph node count		13.8 ± 15.6	9 (1–88)
Pelvic metastatic lymph node count		9.8 ± 10.9	6 (1–65)
Paraaortic metastatic lymph node count		7.2 ± 6.9	5 (1–41)
Ascites volume (cc)		2559 ± 2698.1	2000 (50–18.000)
		**n (%)**	
Lymph node metastasis	Negative	58 (22.5%)	
	Positive	200 (77.5%)	
Metastatic lymph node site	Only pelvic	15 (7.5%)	
	Only paraaortic	52 (26%)	
	Pelvic and paraaortic	124 (62%)	
	Not reported	9 (4.5%)	
Peritoneal cytology	Negative	50 (19.4%)	
	Positive	175 (67.8%)	
	Not reported	33 (12.8%)	
Capsule	Intact	178 (69%)	
	Ruptured	74 (28.7%)	
	Not reported	6 (2.3%)	
Ovarian surface tumor	Negative	17 (6.6%)	
	Positive	235 (91.1%)	
	Not reported	6 (2.3%)	
Uterine serosal tumor	Negative	74 (28.7%)	
	Positive	173 (67.1%)	
	Not reported	11 (4.3%)	
Omental metastasis	Negative	28 (11%)	
	Positive	227 (88%)	
	Not reported	3 (1.2%)	
Diaphragm peritoneal tumor	Negative	249 (96.5%)	
	Positive	7 (2.75%)	
	Not reported	2 (0.8%)	
Ascites	Negative	79 (30.6%)	
	Positive	173 (67.1%)	
	Not reported	6 (2.3%)	
Cytoreduction	Optimal	107 (41.5%)	
	Maximal	151 (58.5%)	

**Table 2:** Factors related with progression-free survival.

Univariate analysis				Multivariate analysis		
Parametre		5-year progression-free survival		Disease failure		
		%	p value	Odds ratio	95%CI	p value
Age^1^	≤50 years	30	0.106			
	>50 years	23				
Total lymph node metastasis	Negative	25	0.626			
	Positive	27				
Pelvic lymph node metastasis	Negative	26	0.403			
	Positive	28				
Paraaortic lymph node metastasis	Negative	30	0.263			
	Positive	26				
Number of remove total lymph node^1^	≤57	24	0.218			
	>57	29				
Number of total metastatic lymph node^1^	≤9	31	0.078			
	>9	21				
Capsule	Intact	24	0.261			
	Ruptured	31				
Uterine serosal invasion	Negative	36	0.114			
	Positive	22				
Peritoneal cytology	Negative	35	**0.026**	1 (Reference)	0.589–1.983	0.803
	Positive	23		1.080		
Omental metastasis	Negative	56	**0.029**	1 (Reference)	0.222–13.196	0.607
	Positive	25		1.710		
Ascites volume^1^	≤2000 cc	37	**<0.0001**	1 (Reference)	1.299–3.328	**0.002**
	>2000 cc	3		2.080		
Cytoreduction	Maximal	35	**<0.0001**	1 (Reference)	1.083–2.777	**0.022**
	Optimal	19		1.735		

1 **:** Median Value

**CI:** Confidence interval

**Table 3:** Factors related with disease-specific survival.

Univariate analysis				Multivariate analysis		
Parametre		5-year disease-specific survival		Death because of disease		
		%	p value	Odds ratio	95%CI	p value
Age^1^	≤50 years	77	0.885			
	>50 years	73				
Total lymph node metastasis	Negative	79	0.250			
	Positive	75				
Pelvic lymph node metastasis	Negative	82	0.122			
	Positive	70				
Paraaortic lymph node metastasis	Negative	79	0.096			
	Positive	75				
Number of removed total lymph node^1^	≤57	74	0.229			
	>57	80				
Number of total metastatic lymph node^1^	≤9	77	0.105			
	>9	70				
Capsule	Intact	75	0.971			
	Ruptured	78				
Peritoneal cytology	Negative	82	0.223			
	Positive	74				
Uterine serosal invasion	Negative	87	**0.038**	1 (Reference)	0.463–3.089	0.712
	Positive	71		1.196		
Omental metastasis	Negative	93	**0.028**	1 (Reference)		0.983
	Positive	74		NC		
Ascites volume^1^	≤2000 cc	77	**0.024**	1 (Reference)	0.885–3.833	0.102
	>2000 cc	58		1.842		
Cytoreduction	Maximal	84	**0.007**	1 (Reference)	1.039–4.904	**0.040**
	Optimal	64		2.257		

1 **:** Median Value

**CI:** Confidence interval

**NC:** Not calculated

## References

[1] Nezhat FR Apostol R Nezhat C Pejovic T New insights in the pathophysiology of ovarian cancer and implications for screening and prevention American Journal of Obstetrics and Gynecology 2015 213 3 262 267 10.1016/j.ajog.2015.03.044 25818671

[2] Mccluggage WG Morphological subtypes of ovarian carcinoma: a review with emphasis on new developments and pathogenesis Pathology 2011 43 5 420 432 10.1097/PAT.0b013e328348a6e7 21716157

[3] Bakkar R Gershenson D Fox P Vu K Zenali M Stage IIIC ovarian/peritoneal serous carcinoma: a heterogeneous group of patients with different prognoses International Journal of Gynecological Pathology 2014 33 3 302 308 10.1097/PGP.0b013e3182988dfd 24681743

[4] Pölcher M Zivanovic O Chi DS Cytoreductive surgery for advanced ovarian cancer Womens Health 2014 10 2 179 190 10.2217/whe.14.4 24601809

[5] Farias-Eisner R Teng F Oliveira M Leuchter R Karlan B The influence of tumor grade, distribution, and extent of carcinomatosis in minimal residual stage III epithelial ovarian cancer after optimal primary cytoreductive surgery Gynecologic Oncology 1994 55 1 108 110 10.1006/gyno.1994.1257 7959250

[6] Gurkan D Ceren Akin A Sahin H Aytac Tohma Y Sahin EA Oncologic outcomes in patients undergoing maximal or optimal cytoreductive surgery for Stage 3C serous ovarian, tubal or peritoneal carcinomas Journal of Obstetrics and Gynaecology 2020 40 4 551 557 10.1080/01443615.2019.1634028 31482736

[7] Eisenhauer EA Therasse P Bogaerts J Schwartz LH Sargent D New response evaluation criteria in solid tumours: revised RECIST guideline (version 1.1) European Journal of Cancer 2009 45 2 228 247 10.1016/j.ejca.2008.10.026 19097774

[8] Eisenhauer EL Abu-Rustum NR Sonoda Y Aghajanian C Barakat RR The effect of maximal surgical cytoreduction on sensitivity to platinum-taxane chemotherapy and subsequent survival in patients with advanced ovarian cancer Gynecol Oncology 2008 108 2 276 281 10.1016/j.ygyno.2007.10.022 18063020

[9] Luyckx M Leblanc E Filleron T Morice P Darai E Maximal cytoreduction in patients with FIGO stage IIIC to stage IV ovarian, fallopian, and peritoneal cancer in day-to-day practice: a Retrospective French Multicentric Study International Journal of Gynecological Cancer 2012 22 8 1337 1343 10.1097/IGC.0b013e31826a3559 22964527

[10] Aletti GD Dowdy SC Gostout BS Jones MB Stanhope CR Aggressive surgical effort and improved survival in advanced-stage ovarian cancer Obstetrics and Gynecology 2006 107 1 77 85 10.1097/01.AOG.0000192407.04428.bb 16394043

[11] Chi DS Franklin CC Levine DA Akselrod F Sabbatini P Improved optimal cytoreduction rates for stages IIIC and IV epithelial ovarian, fallopian tube, and primary peritoneal cancer: a change in surgical approach Gynecologic Oncology 2004 94 3 650 654 10.1016/j.ygyno.2004.01.029 15350354

[12] Chi DS Eisenhauer EL Zivanovic O Sonoda Y Abu-Rustum NR Improved progression-free and overall survival in advanced ovarian cancer as a result of a change in surgical paradigm Gynecologic Oncology 2009 114 1 26 31 10.1016/j.ygyno.2009.03.018 19395008

[13] Eisenhauer EL D’angelica MI Abu-Rustum NR Sonoda Y Jarnagin WR Incidence and management of pleural effusions after diaphragm peritonectomy or resection for advanced mullerian cancer Gynecologic Oncology 2006 103 3 871 877 10.1016/j.ygyno.2006.05.023 16815536

[14] Peiretti M Zanagnolo V Aletti GD Bocciolone L Colombo N Role of maximal primary cytoreductive surgery in patients with advanced epithelial ovarian and tubal cancer: Surgical and oncological outcomes. Single institution experience Gynecologic Oncology 2010 119 2 259 264 10.1016/j.ygyno.2010.07.032 20800269

[15] Wimberger P Lehmann N Kimmig R Burges A Meier W Prognostic factors for complete debulking in advanced ovarian cancer and its impact on survival. An exploratory analysis of a prospectively randomized phase III study of the Arbeitsgemeinschaft Gynaekologische Onkologie Ovarian Cancer Study Group (AGO-OVAR) Gynecologic Oncology 2007 106 1 69 74 10.1016/j.ygyno.2007.02.026 17397910

[16] Eisenkop SM Spirtos NM Friedman RL Lin WC Pisani AL Relative influences of tumor volume before surgery and the cytoreductive outcome on survival for patients with advanced ovarian cancer: a prospective study Gynecologic Oncology 2003 90 2 390 396 10.1016/s0090-8258(03)00278-6 12893206

[17] Bristow RE Tomacruz RS Armstrong DK Trimble EL Montz FJ Survival effect of maximal cytoreductive surgery for advanced ovarian carcinoma during the platinum era: a meta-analysis Journal of Clinical Oncology 2002 20 5 1248 1259 10.1200/jco.2002.20.5.1248 11870167

[18] Hoskins WJ Mcguire WP Brady MF Homesley HD Creasman WT The effect of diameter of largest residual disease on survival after primary cytoreductive surgery in patients with suboptimal residual epithelial ovarian carcinoma American Journal of Obstetrics and Gynecology 1994 170 4 974 979 10.1016/s0002-9378(94)70090-7 8166218

[19] Du Bois A Harter P The role of surgery in advanced and recurrent ovarian cancer Annals of Oncology 2006 17 10 235 240 10.1093/annonc/mdl266 17018731

[20] Gasimli K Braicu EI Nassir M Richter R Babayeva A Lymph Node Involvement Pattern and Survival Differences of FIGO IIIC and FIGO IIIA1 Ovarian Cancer Patients After Primary Complete Tumor Debulking Surgery: A 10-Year Retrospective Analysis of the Tumor Bank Ovarian Cancer Network Annals of Surgical Oncology 2016 23 4 1279 1286 10.1245/s10434-015-4959-4 26832880

[21] Cliby WA Aletti GD Wilson TO Podratz KC Is it justified to classify patients to Stage IIIC epithelial ovarian cancer based on nodal involvement only? Gynecologic Oncology 2006 103 3 797 801 10.1016/j.ygyno.2006.08.047 17052746

